# Bioinformatic Identification of Hub Genes Related to Menopause-Obesity Paradox in Breast Cancer

**DOI:** 10.5812/ijem-140835

**Published:** 2023-11-06

**Authors:** Zahra Hosseinpour, Mostafa Rezaei-Tavirani, Mohammad Esmaeil Akbari

**Affiliations:** 1 Cancer Research Center, Shahid Beheshti University of Medical Sciences, Tehran, Iran; 2 Proteomics Research Center, Faculty of Paramedical Sciences, Shahid Beheshti University of Medical Sciences, Tehran, Iran

**Keywords:** Breast Cancer, Menopausal, BMI, Hub Genes

## Abstract

**Background:**

Breast cancer (BC) is one of the most common cancers in women, significantly contributing to cancer-related death in the modern world. Obesity, as a worldwide epidemic besides the menopausal status, has a paradoxical association with BC.

**Objectives:**

To determine the molecular mechanisms underlying the paradoxical effects of obesity on BC, a comprehensive systems biology analysis was performed.

**Methods:**

Data retrieval, data preprocessing, and differential expression analysis were conducted. Weighted correlation network analysis (WGCNA) identified the gene modules associated with clinical traits. Network analysis and hub gene identification techniques revealed key regulatory genes, and functional enrichment analysis uncovered biological pathways related to hub genes. A logistic regression model was developed to predict menopausal status based on hub genes. Additionally, gene expression analysis of two important genes was performed by qPCR.

**Results:**

The study identified the hub genes and molecular pathways (the PI3K-Akt signaling pathway, proteoglycans in cancer, and lipid metabolic and atherosclerosis pathways) associated with the obesity paradox in BC based on menopausal statutes.

**Conclusions:**

These results may improve our understanding of the underlying mechanisms of the effects of body mass on BC and assist in identifying biomarkers and potential therapeutic targets for treating obese postmenopausal women with BC.

## 1. Background

Breast cancer (BC) is the most common cancer and the leading cause of cancer-related death in women. In 2020, 2.26 million cases of BC were diagnosed worldwide (1). Breast cancer is a complex disease influenced by factors such as body mass index (BMI) and menopausal status. The association between the risk of BC and excess weight is believed to be modified by the menopausal status. Obesity has been suggested to increase BC risk in postmenopausal women (2). However, in premenopausal women, there is a negative correlation between obesity and BC risk, possibly due to hormonal effects. Overweight premenopausal women have lower levels of certain hormones compared to their normal-weight equals (3). The mechanisms underlying these associations are unclear but may involve aromatase activity in non-ovarian body tissues (4). Understanding the interplay between BC cells and adipocytes can provide insights into the relationship between obesity and BC (5).

Gene expression analysis is a powerful tool for unraveling the molecular landscape of BC. This study aims to increase our understanding of the biological mechanisms underlying the protective role of obesity against BC in premenopausal women and its association with BC after menopause. We utilized weighted correlation network analysis (WGCNA) to identify the module genes highly correlated with BMI. The limma package was then used to identify overexpressed genes in obese postmenopausal versus premenopausal women. Hub genes within the upregulated gene module were identified. Additionally, a prediction model was developed to investigate the relationship between the identified hub genes and menopause status using an independent gene expression dataset. Furthermore, gene expression analysis by qPCR was performed. By integrating the results of gene expression analysis, network analysis, prediction modeling, and qPCR, we tried to shed light on the molecular mechanisms linking BMI, menopause, and BC.

## 2. Objectives

Our findings hold the potential for developing personalized therapeutic strategies and identifying novel therapeutic targets in BC.

## 3. Methods

### 3.1. Data Retrieval

The gene expression dataset, GSE24185 (ID: 130113), was obtained from the GEO database using the R package GEOquery (v.2.68.0) (6), including gene expression data from primary breast tumor samples with a focus on the relationship between BMI, menopause, and BC. The dataset was generated on 18 Jul 2011 using the Agilent GPL96 platform (HG-U133A) and consisted of the expression profiles of 22,278 genes (7). Additionally, the TCGA-BRCA dataset, which was accessed through the TCGAbiolinks R package (v.2.28.3) (8), was the source for genomic and clinical data from BC patients, including gene expression data and menopausal status. Preprocessing was performed to ensure the quality and compatibility of data for subsequent analyses.

### 3.2. Differential Expression Analysis

Differential expression analysis was conducted to identify the genes differentially expressed in the PRE- and POST-menopause subtypes within normal-weight and overweight BMI categories. The limma package (8) was used along with the criteria of |log2foldchange| > 1 and an adjusted P-value < 0.05 to define significant fold changes. Gene expression data from GSE24185 were preprocessed, normalized, and fitted to a linear model considering menopause status and BMI category as factors. Empirical Bayes moderation improved gene-specific variance estimation. Contrasts were defined to compare PRE- and POST-menopause subtypes within each BMI category, and moderated t-statistics, log-fold changes, and adjusted P-values were computed using the eBayes function. The DEGs that met these criteria were identified as significantly upregulated or downregulated. Separate analyses for normal-weight and overweight BMI categories yielded two sets of DEGs.

### 3.3. Construction of Weighted Correlation Network Analysis

Gene co-expression patterns associated with clinical traits were explored using the WGCNA (v. 1.72-1) approach, a widely used tool in systems biology (9) used to analyze co-expression networks and to eliminate genes or samples with missing values and outliers (based on a prespecified height cut). The "PickSoftThreshold" function determined the appropriate soft thresholding power (β) to construct the weighted adjacency matrix based on a high scale-free topology fit index. The weighted adjacency matrix was transformed into the topological overlap matrix (TOM), and the dissimilarity of TOM (diss TOM) was calculated using the "hclust" function, generating a hierarchical clustering tree (dendrogram) that could assign highly interconnected genes to the same module.

### 3.4. Module-Trait Relationships

Module eigengenes (MEs), representing summary weighted averages of module gene expression profiles, were determined using the "module eigengenes" function. The association between MEs and clinical traits (i.e., BMI, menopause, and age) was analyzed using the "corPvalueStudent" function. A heat map plot showing the relationships between 12 modules and clinical traits was generated, considering a statistical significance threshold of P < 0.05. In summary, WGCNA was employed to construct co-expression networks and identify modules with highly interconnected genes. The relationship between these modules and clinical traits was assessed using module eigengenes.

### 3.5. Network Construction and Hub-Gene Identification

The STRINGdb package (v.2.12.1) was utilized to retrieve protein-protein interaction data (10) and construct the network for the desired module of upregulated genes. Interactions with a score threshold of 200 were considered. Network analysis techniques were applied to identify hub genes (i.e., highly connected and influential genes within the network). Node centrality parameters, such as degree, betweenness centrality, and closeness centrality, were computed using the igraph package (v.1.5.1) (11). The hub genes identified within the desired module were further enriched by utilizing the KEGG package (v.1.60.0) (12). This enrichment analysis provided insights into the potential roles and functions of the hub genes within the context of the upregulated gene module.

### 3.6. Prediction Model Development

A prediction model was developed using the caret package to explore the relationship between menopause status and the hub genes within the turquoise module (13). The model’s performance was evaluated using the TCGA-BRCA dataset, which includes gene expression data and clinical information from BC patients. The Durbin-Watson test was utilized to assess the independence assumption and examine autocorrelation in residuals. A correlation matrix was generated to determine highly correlated features in a given dataset, and those above the 0.7 threshold were removed to address multicollinearity. After feature selection, a binary logistic regression model was built using the filtered dataset designating non-correlated hub genes' expression levels as independent variables and menopause status as the dependent variable. A logistic regression algorithm was employed to estimate coefficients. Standard evaluation metrics, including accuracy, precision, recall, and area under the curve (AUC)-receiver operating characteristic (ROC), were created to assess the model’s classification performance. These metrics provided valuable insights into the model’s accuracy in predicting menopause status and distinguishing between classes (14). All statistical analyses and model development were conducted in R software (v. 4.3.0) (15).

### 3.7. RNA Extraction and cDNA Synthesis

First, the BMIs of the patients were calculated. Peripheral blood samples were then collected from three BC patients (i.e., the case group) and three healthy subjects (i.e., the control group) to extract total RNA using TRIzol reagent (Invitrogen, Germany). The quality and quantity of the extracted RNA were assessed using a BioPhotometer (Eppendorf, Germany) and gel electrophoresis. For cDNA synthesis, 1 μg of RNA was utilized, and the AmpliSens^®^ AII-screen-FRT PCR kit (Exiqon, Denmark) was used following the manufacturer’s thermal protocol. The synthesis process involved incubation at 42°C for 60 minutes, followed by a 5-minute incubation at 95°C.

### 3.8. Gene Expression Analysis by qPCR

Locked nucleic acid (LNA) specific primer sets (Exiqon, Denmark) were utilized for quantifying the OSBPL7 and KIR2DL2 genes, whose relative expression levels were normalized to the BET endogenous reference gene using the 2^-ΔΔCT^ method. Real-time PCR reactions were conducted using a real-time PCR kit (Takara Bio, Japan) in the Chromo4™ System (Bio-Rad, USA). All samples were run in technical triplicates for 40 cycles with the following thermal cycling conditions: Pre-denaturation at 95°C for 10 minutes, denaturation at 95°C for 20 seconds, annealing at 60°C for 30 seconds, and extension at 72°C for 30 seconds. The statistical significance of the differences between the control and cancer groups was assessed using an F test, and relevant graphs were generated using GraphPad Prism 9. The primer sequences used in this study are mentioned in Table 1. 

**Table 1.** Primer Information

Primer Name	Primer Sequence (Base)	Primer Length (Base)
**f-OSBPL7**	GAGGCTTCCGCTTCATCAGT	20
**r-OSBPL7**	TTCCCACAGGCACAATCTCC	20
**f-KIR2DL2**	CACCCACTGAACCAAGCTCT	20
**r-KIR2DL2**	ACCAGCGATGAAGGAGAAAG	20

## 4. Results

This analysis focused on the correlation of gene transcription profiles of human breast tumors with patients’ BMIs. Table 2 provides a summary of patients’ information and tumor attributes, encompassing factors such as obesity, race, and menopausal status. Obesity status was defined according to WHO’s BMI categories as normal/lean (B24.9), overweight (BMI = 25 - 29.9), and obese (C30).

**Table 2.** Phenotypic Characteristics of Patients (n = 103)

	Mean ± SD or No. (%)
**Age**	49.03 ± 9.35
**BMI**	
Normal	36 (35.0)
Obese	38 (36.9)
Overweight	29 (28.2)
**Menopause**	
PERI	9 (8.7)
POST	45 (43.7)
PRE	49 (47.6)
**Race**	
Asia	10 (9.7)
Black	16 (15.5)
White	77 (74.8)

Abbreviation: BMI, body mass index.

### 4.1. Differential Expression Analysis

Table 3 presents a comprehensive list of DEGs, including their log-fold changes (logFC) and P-values, obtained through differential expression analysis. This table provides detailed information on the genes that exhibited statistically significant differences in their expression profiles between the PRE-and POST-menopause subtypes within overweight BMI categories.

**Table 3.** Differentially Expressed Genes with Respective Log-Fold Changes and P-Values

Gene Symbol	LogFC	P-Value
**KIR2DL2**	2.377429	0.002221
**UPK3A**	2.078524	0.001733
**OSBPL7**	2.014238	0.086029
**RAB11FIP3**	1.99	0.013999
**EPHB2**	1.980095	0.024103
**SLC7A1**	1.853714	0.030629
**CYP2B7P///CYP2B6**	1.704286	0.08639
**KIR2DL1**	1.654048	0.004174
**STK11**	1.621762	0.004045
**LOC101928551///ADAM29**	1.581762	0.018565
**KCNK10**	1.533857	0.052136
**FOXB1**	1.521857	0.074973
**MOCS1**	1.40981	0.015247
**SDK2**	1.361333	0.041162
**PCK1**	1.359667	0.027364
**KCND1**	1.341619	0.008729
**CYP2B7P**	1.326429	0.064255
**EML2**	1.271571	0.007609
**MAOB**	1.256048	0.034949
**PPP1R37**	1.236191	0.039397
**PLAC4**	1.227333	0.001813
**FABP4**	1.150429	0.20874
**SOX1**	1.140714	0.113474
**SNX1**	1.129238	0.082378
**KRT84**	1.122143	0.030868
**RRBP1**	1.119095	0.016515
**CALCA**	1.105143	0.028907
**TEC**	1.093381	0.002228
**EGFR**	1.057191	0.012544
**ADAM3B**	1.048	0.028067
**FETUB**	1.031286	0.091751
**CLSPN**	1.030333	0.19274
**FGG**	1.017667	0.059438
**CA12**	1.010952	0.107776
**FGB**	1.004286	0.032576
**MGP**	1.002571	0.107937

Abbreviation: LogFC, log-fold changes.

### 4.2. Construction of Co-expression Modules

**Figure 1.**
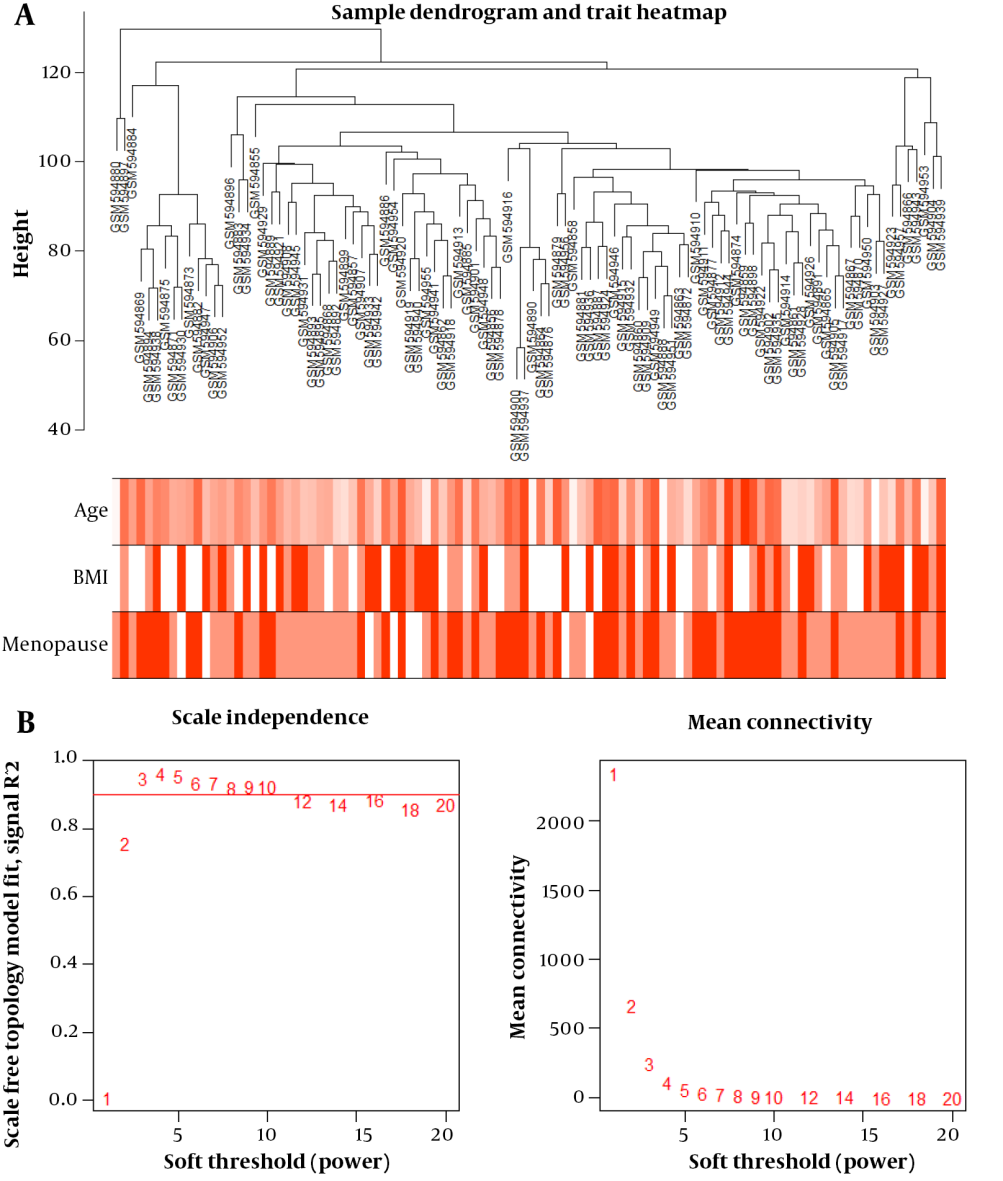
A, sample dendrogram and the heatmaps of trait indicators. Color intensity was proportional to age, body mass index (BMI), and menopause status; B, analysis of scale-free and mean connectivity for various soft-thresholding powers

Cluster analysis with the FlashClust tools package identified one outlier sample, which was subsequently removed (Figure 1A). The appropriate soft-threshold (power) value for network construction was determined by evaluating scale-free and mean connectivity across various power values. A power value of 8 was selected as the optimal threshold (Figure 1B). The dendrogram revealed 12 distinct modules whose genes exhibited similar co-expression traits. These co-expression modules provided insights into the coordinated expression patterns of the genes associated with obesity, menopause status, and age, contributing to our understanding of the molecular mechanisms and potential biomarkers related to different obesity phenotypes.

### 4.3. Correlation Between Modules and Clinical Traits

**Figure 2.**
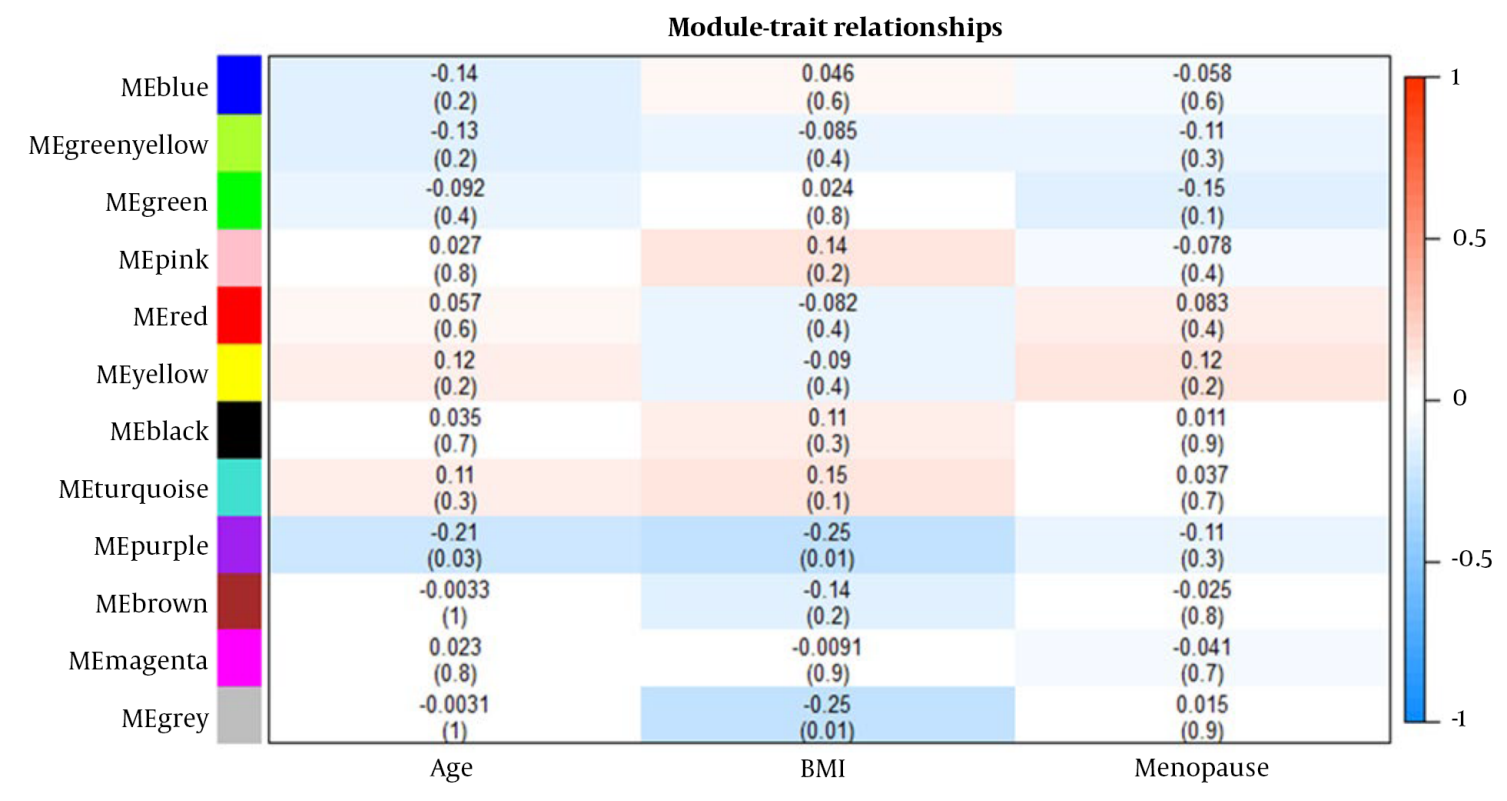
Module and trait-related heat maps. Horizontal coordinates represent traits (age, body mass index (BMI), and menopause status). Vertical coordinates are the clusters obtained for the modules.

The associations between modules and clinical traits were analyzed by correlating eigengenes with the respective traits, leading to the identification of significant correlations (Figure 2). The blue, pink, black, and turquoise modules exhibited the strongest positive correlation with BMI, while the red, yellow, and turquoise modules showed correlations with age. The red and yellow modules displayed the highest positive correlation with menopause. Given that the turquoise module contained common differentially expressed genes (DEGs), it was chosen for further analysis.

### 4.4. Identification of Hub Genes and Enrichment Analysis

After network analysis, the top hub genes within the turquoise module were identified based on their high connectivity and influence. The 30 most significant hub genes in the module were as follows: *OSBPL7*, *SNX13*, *SDK2*, *MAOB*, *CA12*, *FETUB*, *RAB11FIP3*, *CLSPN*, *UPK3A*, *KCNK10*, *KCND1*, *PPP1R37*, *CALCA*, *MGP*, *STK11*, *PCK1*, *MOCS1*, *KIR2DL1*, *EML2*, *RRBP1*, *EPHB2*, *TEC*, *SLC7A1*, *EGFR*, *KRT84*, *ADAM29*, *FABP4*, *FGB*, *FOXB1*, *CYP2B6*, and *PLAC4*. To gain insights into the functional significance of these hub genes, enrichment analysis was performed using the KEGG package (v.1.60.0). This analysis identified the dominant biological pathways associated with these hub genes (Table 4). 

**Table 4.** The Significant Biological Pathways Associated with Hub Genes

Pathways	Hub Genes	Enrichment P-Value	Adjusted P-Value (P. Adjust)
**PI3K-Akt signaling pathway (hsa04151)**	*OSBPL7, SNX13, SDK2, MAOB, CA12, FETUB, RAB11FIP3, CLSPN, UPK3A, KCNK10*	1.43E-07	3.61E-06
**Cancer-related proteoglycans (hsa05205)**	*OSBPL7, SNX13, SDK2, CA12, FETUB, RAB11FIP3, CLSPN, UPK3A, KCNK10*	1.41E-08	1.43E-06
**Lipid metabolism and atherosclerosis (hsa05417)**	*OSBPL7, SNX13, SDK2, CA12, FETUB, RAB11FIP3, CLSPN, UPK3A, KCNK10*	2.14E-08	1.44E-06

### 4.5. Logistic Regression Model Performance

**Figure 3.**
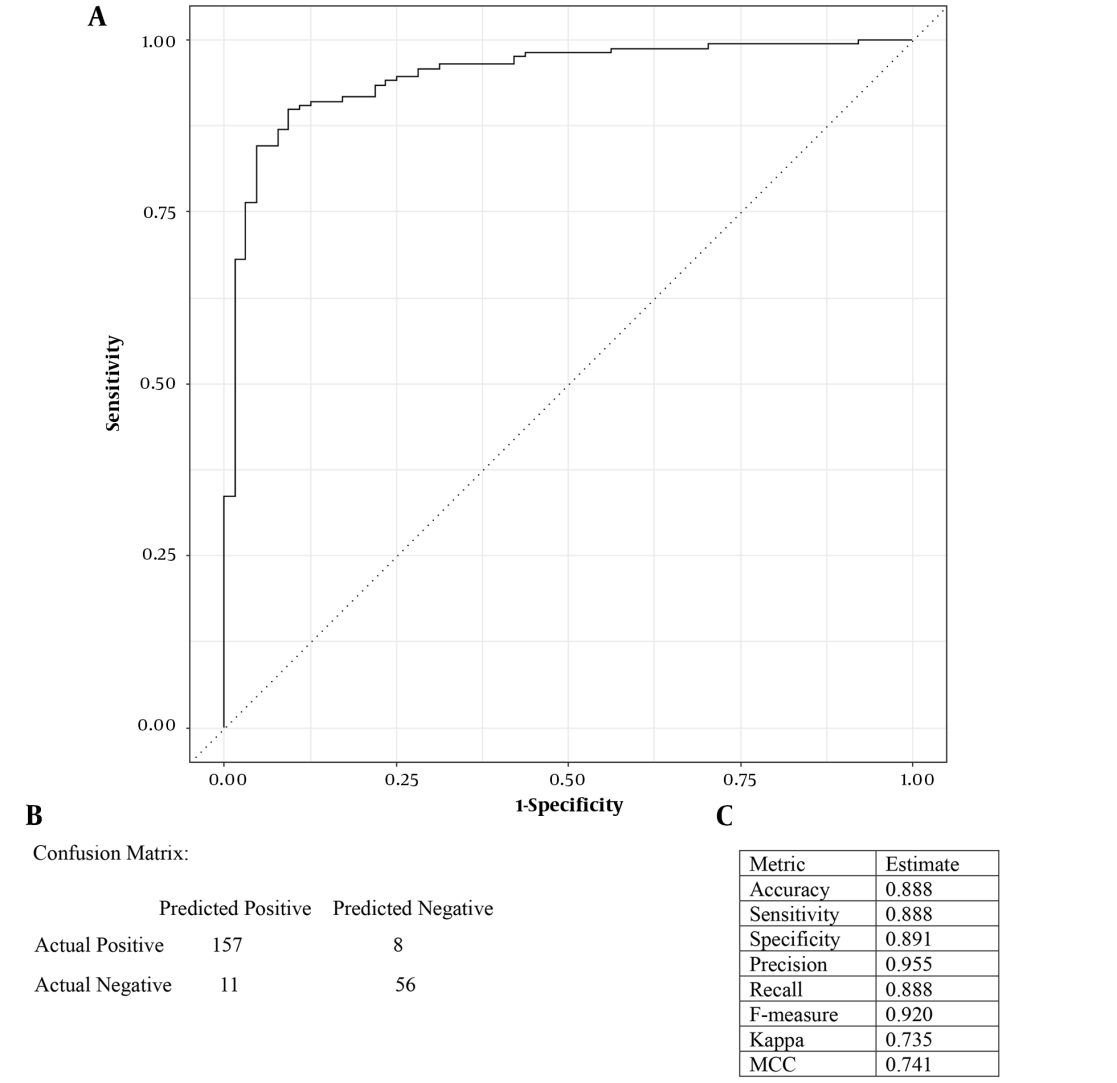
A, receiver operating characteristic (ROC) curve depicting the area under the curve (AUC) analysis. The x-axis represents the false positive rate (1-specificity), while the y-axis represents the true positive rate (sensitivity). The closer the AUC value is to 1, the higher the predictive ability of the model; B, confusion matrix of the logistic regression model; C, performance metrics of the logistic regression model

This study aimed to identify menopause-associated genes using gene expression analysis and a binary logistic regression approach based on a dataset of selected hub genes. The computed D-W statistic was equal to 1.761726 (P-value = 0), providing evidence against the null hypothesis (i.e., no autocorrelation in residuals). Highly correlated features were identified through correlation analysis, and the "FGG" feature was removed to address multicollinearity. After feature selection, a logistic regression model was constructed using the filtered TCGA-BRCA dataset. Model performance was evaluated through 10-fold stratified cross-validation, demonstrating an AUC value verifying the model’s discrimination ability for positive and negative cases (Figure 3A). The model achieved an accuracy of 0.888, meaning that it could correctly classify 88.8% of the cases. Also, the sensitivity and recall values of 0.888 indicated the successful identification of menopausal women. Logistic regression coefficients provided insights into these genes’ contributions to menopause prediction, where a set of these genes were significantly associated with menopause status, suggesting their involvement in menopause-related biological processes.

### 4.6. Real-Time PCR

**Figure 4.**
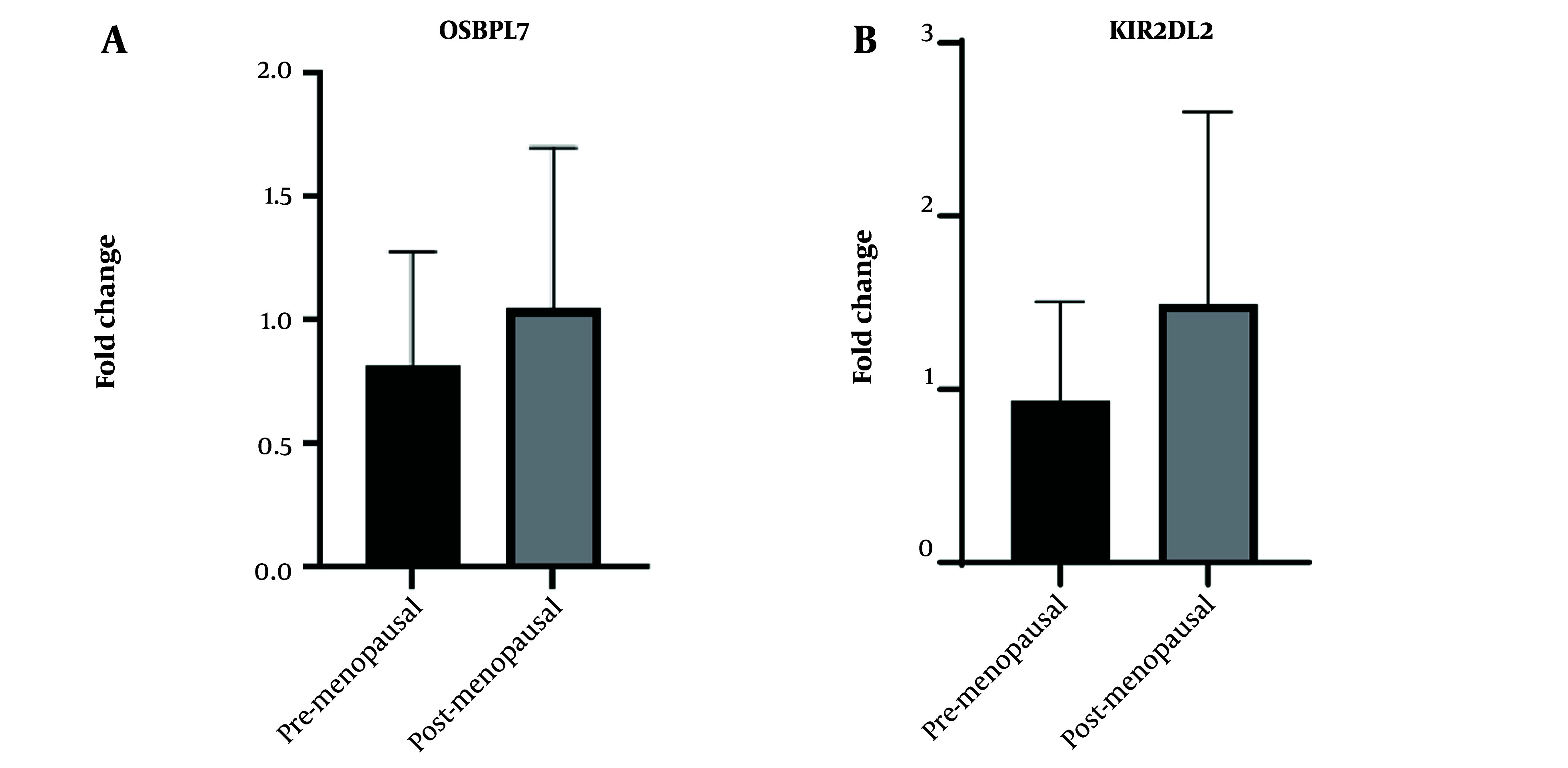
The comparative expression analysis of the *KIR2DL2* and *OSBPL7* genes between obese postmenopausal women vs. normal weight premenopausal women

The mRNA expression analysis of *KIR2DL2* in obsess postmenopausal versus normal weight premenopausal individuals (Figure 4), as determined by an unpaired *t*-test, yielded no statistically significant difference between the two groups (P-value = 0.6507). The mean expression level of *KIR2DL2* in premenopausal individuals was 0.8214, while in postmenopausal individuals, it was 1.046. The difference in means (B-A) was 0.2244 ± 0.4593 SEM, with a 95% confidence interval ranging from -1.051 to 1.500. Additionally, an F test for variance comparison further supported the absence of a significant variance difference between the two groups (P = 0.6556). Similarly, the analysis of *OSBPL7* mRNA expression showed no statistically significant difference between postmenopausal and premenopausal individuals (P-value = 0.4842). The mean expression levels of *OSBPL7* in premenopausal and postmenopausal individuals were 0.9349 and 1.49, respectively, with a mean difference of 0.5554 ± 0.7211 SEM and a 95% confidence interval from -1.447 to 2.558. The F test for variance analysis also indicated no significant difference between the two groups (P-value = 0.4166). It is important to note that both analyses were conducted on a relatively small sample size (n = 3 in each group), and given the limited sample size, these results collectively suggested that neither *KIR2DL2* nor *OSBPL7* mRNA expression levels exhibited significant differences between premenopausal and postmenopausal individuals.

## 5. Discussion

This study utilized a comprehensive bioinformatic analysis to investigate the molecular mechanisms underlying the paradoxical role of obesity in modifying the risk of BC according to menopausal status. The analysis identified hub genes, including *OSBPL7*, *SNX13*, *SDK2*, *MAOB*, *CA12*, *FETUB*, *RAB11FIP3*, *CLSPN*, *UPK3A*, *KCNK10*, *KCND1*, *PPP1R37*, *CALCA*, *MGP*, *STK11*, *PCK1*, *MOCS1*, *KIR2DL1*, *EML2*, *RRBP1*, *EPHB2*, *TEC*, *SLC7A1*, *EGFR*, *KRT84*, *ADAM29*, *FABP4*, *FGB*, *FOXB1*, *CYP2B6*, and *PLAC4*, which showed significant differential expression in obese and non-obese groups and distinct expression patterns in premenopausal and postmenopausal subgroups. Functional enrichment analysis revealed several biological pathways related to the hub genes identified within the gene modules. Among these pathways, the PI3K-Akt signaling pathway, cancer-related proteoglycans, and lipid and atherosclerotic pathways were of particular interest. The PI3K-Akt signaling pathway regulates cells’ survival, growth, and proliferation, and its dysregulation in BC leads to uncontrolled cell growth and tumor progression (16). Proteoglycans, found in the extracellular matrix, are implicated in cancer progression. In BC, these molecules interact with the components of the PI3K-Akt pathway, activating oncogenic signaling cascades that promote tumor growth, invasion, and metastasis (17). Dysregulated lipid metabolism is associated with increased BC risk and poor prognosis. In summary, the PI3K-Akt pathway connects proteoglycans, lipid metabolic routes, atherosclerosis, and BC. Perturbations in lipid metabolism, for example, in atherosclerosis, may lead to BC development (18, 19). In addition to pathway enrichment analysis, two specific genes, *KIR2DL1* and *OSBPL7*, were identified to be upregulated. Pathway analysis of these overexpressed genes identified that the *OSBPL7* gene was involved in the synthesis of bile acids and bile salts and the metabolism of steroids, while the *KIR2DL2* gene was engaged in immunoregulatory interactions between lymphoid and non-lymphoid cells.

Clinical and epidemiological studies have shown that the prevalence of cholesterol gallstone disease is twice as high in women compared to men (20). Many studies have established that postmenopausal hormone replacement therapy (HRT) increases the risk of BC and gallstone formation (21). Bile acids comprise the largest family of steroidal mediators that, at relatively low concentrations, regulate metabolic processes by binding to Farnesoid-X-receptors (FXR) and G protein bile acid-activated receptor (GPBAR)-1 (22), also known as Takeda G-protein-coupled receptor 5 (TGR5) (23). At high concentrations, these steroidal mediators activate both membrane and nuclear receptors (24). Immune escape is an important hallmark of cancer, contributing to tumor progression and metastasis. Natural killer (NK) cells play crucial roles in protective immunity against tumors (25). Obesity is associated with alterations in NK cell phenotype (26). These cells have inhibitory and stimulatory surface receptors that control their activation. Inhibitory receptors, such as KIR2DL1, KIR2DL2, and KIR2DL3, bind to HLA-C on target cells, regulating NK cell cytotoxicity and cytokine production (27). In this study, KIR2DL2, a defining gene of the KIR B-haplotype, was upregulated in postmenopausal overweight women. Similar findings were reported by Jobim et al., who found a strong association between KIR2DL2 and BC in Brazilian women (28).

This study has limitations that should be acknowledged. The small sample size is a notable limitation, potentially impacting the generalizability of the results. Larger and more diverse cohorts should be considered in future studies to obtain more generalizable findings. Another limitation is the reliance on publicly available datasets and gene expression data alone without considering other potential influencing factors, such as genetic variations, lifestyle, and environmental factors, which should be addressed in future research to provide a more comprehensive understanding of the relationship between BMI, menopausal status, and BC. The predictive model used in this study also had shortcomings. While it demonstrated significant predictive ability, the accuracy of the model might have been influenced by the complexity and multifactorial nature of menopausal status. In order to enhance the model’s predictive power, future studies should consider including additional factors like genetic variations and lifestyle elements. Despite these limitations, this study provides valuable insights into the molecular mechanisms connecting BMI, menopausal status, and BC. We were able to identify important hub genes and explore the key molecular pathways, helping expand our understanding of BC development in relation to BMI and menopausal status. To enhance the validity of these findings and expand our understanding of BC, it is important to validate these results and explore additional datasets. Future studies should include larger sample sizes, incorporate additional factors, and utilize more diverse datasets to obtain more robust and generalizable conclusions.

### 5.1. Conclusions

In conclusion, this study sheds light on the intricate relationship between BMI, menopausal status, and risk of BC at the molecular level. Despite the above-mentioned limitations, our findings provide valuable insights into the molecular mechanisms underlying BC development in relation to BMI and menopausal status. More research is warranted to validate these findings by considering additional factors and to advance our knowledge of BC, facilitating the development of targeted therapies.
